# Bradycardia Associated with Steroid Use for Laryngeal Edema in an Adult: A Case Report and Literature Review

**DOI:** 10.1155/2016/9785467

**Published:** 2016-11-24

**Authors:** Preeti R. John, Ariana Khaladj-Ghom, Kimberly L. Still

**Affiliations:** ^1^Baltimore VA Medical Center, Baltimore, MD, USA; ^2^Department of Surgery, University of Maryland Medical Center, Baltimore, MD, USA; ^3^Baltimore VA Medical Center, Surgical Intensive Care Unit, 10 North Greene Street, 5C-119, Baltimore, MD 21201, USA; ^4^University of Maryland Medical Center, 16 S. Eutaw, Suite 500, Baltimore MD 21201, USA

## Abstract

Steroids are used for specific indications in the perioperative period to reduce laryngeal or spinal cord edema, or for prophylaxis and treatment of postoperative nausea and vomiting. Given the other potential causes for hemodynamic alterations in the perioperative setting, it is important for physicians to be aware of cardiovascular side effects of short term steroids. Changes in blood pressure and heart rate, cardiac dysrhythmias, and even death have been described in patients receiving short term intravenous steroids. Bradycardia has been reported following short term methylprednisolone and dexamethasone therapy in both adult and pediatric patients. There are only two case reports in the literature of bradycardia following short term intravenous dexamethasone use in adult patients. This is the first case report that describes bradycardia following the use of dexamethasone in the postoperative setting for management of laryngeal edema in an adult. Telemetry and twelve lead electrocardiograms revealed sinus bradycardia and correlated directly with administration of dexamethasone in our patient. Bradycardia resolved following discontinuation of dexamethasone. We advocate for hemodynamic monitoring in patients receiving more than one dose of intravenous steroid therapy in the perioperative period, especially those with known cardiac and hepatic comorbidities and those taking medications with negative chronotropic effects.

## 1. Introduction

Short term steroids are used frequently for a variety of indications in the perioperative period. In patients undergoing elective spine surgery, steroids are used to reduce spinal cord edema [1]. In traumatic spinal cord injury, steroids have been shown to confer significant recovery of motor function [2]. Similarly in patients undergoing repair of thoracic and thoracoabdominal aortic aneurysms, intravenous steroids and CSF drainage are used as adjunctive measures to prevent spinal cord ischemia [3]. Following intubation, intravenous steroids are often administered to reduce laryngeal edema prior to extubation [4]. Dexamethasone is used for prophylaxis and treatment of postoperative nausea and vomiting [5].

Given the frequent use of steroids and the other potential causes for hemodynamic alterations in the perioperative setting, it is important for physicians to be aware of cardiovascular side effects of short term steroids. Changes in blood pressure and heart rate, cardiac dysrhythmias, and even death have been described in patients receiving short term intravenous steroids [6–9]. Bradycardia has been reported following short term methylprednisolone therapy in both adult and pediatric patients [10–15]. To our knowledge there are only two case reports in the literature describing bradycardia following short term intravenous dexamethasone use in adult females. In both cases, bradycardia was self-limiting and resolved after steroid discontinuation [16, 17].

This is the first case report describing bradycardia secondary to dexamethasone use for airway edema in an adult.

The aim of this case report and literature review is to increase awareness of the adverse cardiovascular effects of short term intravenous steroids. Hemodynamic monitoring should be considered in patients receiving short term steroids, particularly those patients with coexisting cardiac and liver disease and those receiving other drugs with negative chronotropic effects.

## 2. Case Presentation

A 58-year-old male presented to the hospital with mesh infection at the site of a previous ventral hernia repair. Past medical history was significant for hypertension, mild aortic stenosis, type two diabetes mellitus, morbid obesity with body mass index (BMI) 48.8, and severe obstructive sleep apnea for which he utilized continuous positive airway pressure (CPAP) ventilation. He had normal liver and kidney function. His physical status was determined to be 3 by the American Society of Anesthesiologists (ASA) Physical Status classification system.

He was taken to the operating room to undergo surgery under general anesthesia. The procedure involved explantation of infected abdominal wall mesh and excision of a sinus tract with small bowel resection and lysis of adhesions. Preinduction vitals were as follows: blood pressure of 137/81 mmHg, heart rate of 86 beats per minute (BPM), oxygen saturation of 95% on room air, and temperature of 36.8 degrees Celsius. The surgery lasted 5 hours, with estimated blood loss of 150 mL and no intraoperative hemodynamic instability.

Orotracheal intubation for surgery required several attempts. First attempt under direct laryngoscopy revealed a grade 3 view (Cormack-Lehane classification of laryngeal view). Second attempt with a fiber-optic device was aborted because of copious secretions and poor view. Third attempt was with a Glidescope, with grade 1 view of the vocal cords, resulting in successful intubation.

Given the multiple attempts at intubation, there was concern for significant airway edema. In the setting of severe sleep apnea and morbid obesity, the decision was made to keep the patient intubated postoperatively. Because of fevers, thick secretions, and a low PaO_2_ : FiO_2_ ratio of 222, he remained intubated until postoperative day (POD) 3. Dexamethasone was started prior to extubation (10 mg intravenously every six hours) in anticipation of persistent airway edema.

On POD 3, he was extubated following a successful pressure support trial. However, during the following thirty minutes, he demonstrated increased work of breathing with audible stridor. Noninvasive ventilation was attempted unsuccessfully, and he eventually required endotracheal intubation for hypercarbic respiratory failure. Glidescope visualization during reintubation revealed a significantly swollen epiglottis and vocal cords. Otolaryngologists were consulted and the decision was made to continue dexamethasone to reduce laryngeal edema.

Within twenty-four hours of the first dose of dexamethasone, telemetry demonstrated the patient's heart rate dropping from 30 to 60 BPM. The patient was asymptomatic and the rhythm was sinus bradycardia, not associated with hypotension. Serial troponins and 12 lead electrocardiograms (ECGs) were obtained and were negative for acute coronary syndrome. Other etiologies for bradycardia such as vasovagal reflex and electrolyte abnormalities were excluded. No other negative inotropic or chronotropic medications (such as beta blockers, nondihydropyridine calcium channel blockers, digoxin, or amiodarone) were concomitantly administered.

After a two-day course of dexamethasone, the patient was successfully extubated on POD 5. On POD 6, dexamethasone was discontinued. His heart rate returned to baseline by POD 7.

On the night of POD 7, the patient was noncompliant with noninvasive ventilation (CPAP machine). He decompensated and was reintubated for hypercarbic respiratory failure. Arterial blood gas revealed respiratory acidosis (ABG: pH7.21/PCO2 100 mmHg/PO2 106 mmHg). He was in normal sinus rhythm with heart rates ranging from 60 to 80 BPM. Chest X-ray did not reveal pulmonary edema or infiltrate, but sputum cultures were positive for pan-sensitive* Staphylococcus aureus*, and he was treated with a course of nafcillin.

On POD 9, the patient remained intubated and intravenous dexamethasone (10 mg every 6 hours) was restarted because of concern for laryngeal edema. This continued for fifteen doses over the next four days. On POD 10, sinus bradycardia became evident again, with heart rate of 30–50 BPM. All medications were examined for bradycardic effect and the only one identified to have the potential for negative chronotropy was propofol, which was discontinued. Midazolam was used instead (for sedation), while he remained on ventilator support.

In spite of discontinuation of propofol, the bradycardia persisted and bigeminy developed on the night of POD 12. The lowest heart rate recorded was 31 BPM. Dexamethasone was discontinued on the morning of POD13 (last dose 06:00), with subsequent resolution of bradycardia within twelve hours. No other medication changes were made on that day.

On POD 14, the patient went to the operating room for direct laryngoscopy and tracheostomy. He was eventually weaned to tracheostomy collar and ultimately discharged home on POD 28, clinically stable and ambulating with tracheostomy collar in place. For the remainder of his hospital course, his heart rate remained within normal limits, 70–80 BPM (see Table 1 and 12 lead EKG).

## 3. Discussion

Significant hemodynamic alterations secondary to short term steroids have been reported [18]. Bradycardia (defined as a heart rate less than 60 BPM) has been described with high-dose pulse methylprednisolone therapy used in autoimmune diseases [19]. It has also been described following short term oral prednisone use [20, 21].

Cardiac arrhythmias as a result of dexamethasone use have been less frequently described and primarily involve pediatric patients [11, 22]. To our knowledge, there are only two case reports of dexamethasone-induced cardiac dysrhythmia in adult patients. One report described bradycardia in an adult female who received a 4 mg dose of intravenous dexamethasone upon induction of anesthesia for prophylaxis of postoperative nausea and vomiting [16]. The other case report describes frequent premature ventricular contractions (PVCs) in an adult female receiving dexamethasone postoperatively following cardiopulmonary bypass for aortic and mitral valve replacement [17].

This is the first case report describing sinus bradycardia in an adult male patient following dexamethasone use for laryngeal edema.

The strong temporal relationship, including positive dechallenge and rechallenge, highly suggests dexamethasone as the etiology of the patient's bradycardia. The Naranjo scale is a tool used to estimate the probability of an adverse drug event, with scores ranging from 0 to 13 and a higher score suggesting a stronger probability [23]. Using this scale, we calculated a score of 9, indicating a high probability of an adverse drug event (see Table 2).

The mechanism of steroid-induced dysrhythmia is not known, but several hypotheses have been suggested. Steroids have intrinsic mineralocorticoid activity that can cause sodium retention and subsequent hypertension. Baroreceptor-mediated reflex bradycardia in response to hypertension is a potential explanation. However, although our patient had a history of hypertension, his blood pressure was not elevated during the postoperative period.

Blunting of the chronotropic response to catecholamines is another potential mechanism of bradycardia development. In an animal model, Hall et al. showed depression of cardiovascular alpha and beta adrenergic receptor sensitivity with high-dose steroid therapy [24]. This theory is plausible in our patient, who was receiving high-dose dexamethasone at a rather short interval over several days.

Steroids play a role in altering potassium flux across the cell membrane, and steroid-induced cardiac abnormalities may be a consequence of sudden changes in potassium flux across the cell membrane. Serum potassium levels and fractional excretion of potassium increased from baseline after treatment with pulse steroid therapy in a study by Fujimoto et al. Transient shifts in renal electrolyte excretion after intravenous methylprednisolone administration were proposed to cause shifts across myocardial cell membranes, causing cardiac dysrhythmias [25]. For these reasons, electrolyte monitoring during steroid treatment is prudent, especially in patients with cardiac and liver disease and those with compromised skin integrity who may be predisposed to electrolyte shifts, as in burns and dermatologic conditions (such as pemphigus, toxic epidermal necrolysis, and erythroderma) [9].

Transient, direct damage to the myocardium has also been suggested as a possible cause of steroid-induced cardiac dysrhythmias [15].

The preparations with which the steroid injections are administered must also be considered. For example, phosphate buffered solutions may lead to formation of calcium phosphate complexes, causing sudden alterations in ionized calcium levels that may potentiate arrhythmias [17]. Our patient received dexamethasone sodium phosphate 4 mg/mL injection manufactured by Fresenius Kabi USA, LLC.

Another consideration is the hepatic metabolism of dexamethasone. The liver is primarily responsible for metabolism of this drug to inactive metabolites which are then excreted in the urine. In patients with hepatic insufficiency, it has the potential to accumulate [26]. Dexamethasone is a long acting glucocorticoid, with a biological half-life of 36–54 hours. Despite normal liver function in our patient, the long biological half-life and frequent dosing of dexamethasone in our patient may have led to increasing plasma levels and subsequent bradycardia. The onset of action of dexamethasone is 8–24 hours, which is probably why the bradycardia did not manifest immediately after administration of the first dose of the drug [27].

Finally, anaphylaxis to steroids may be a potential mechanism for adverse cardiovascular reactions [28], though one that is likely to be recognized immediately. Our patient did not exhibit any significant signs or symptoms to suggest anaphylaxis (i.e., urticaria, hypotension, or shock).

## 4. Conclusions

Steroids are used frequently in surgical patients for indications such as upper airway edema and spinal cord edema. Given the other potential causes for hemodynamic alterations in the perioperative setting, it is important to be aware of all potential cardiovascular side effects of steroids, including bradycardia, a rare but potentially serious adverse effect.

Specific treatment is not warranted in asymptomatic sinus bradycardia, as it is usually self-limiting and resolves after discontinuation of dexamethasone. However, there have been reports of cardiac dysrhythmias with hemodynamic instability and even death following short term intravenous steroid use.

We advocate for consideration of hemodynamic monitoring in patients receiving more than one dose of intravenous steroid therapy in the perioperative setting. The need for monitoring should be determined based on the patient's active and past medical problems, including cardiac and hepatic comorbidities, concomitant drug therapy (negative chronotropes), and any previous adverse reactions to steroids. When steroids are determined to be the implicating agent, the risks and benefits should be considered and when possible, this drug should be discontinued.

## Supplementary Material

Electrocardiograms obtained following steroid administration show the following:Sinus bradycardia;
Sinus bradycardia with frequent premature ventricular contractions in a pattern of bigeminy.

## Figures and Tables

**Table 1:** Sequence of postoperative events and dexamethasone administration.

Postoperative day	Event	Dexamethasone	Heart rate
0	Abdominal surgery		80 s
1			90 s
2			80 s–90 s
3	Extubated, reintubated	First dose, 6 am	70 s–90 s
4			30 s–60 s
5	Extubated	Last dose, 6 am	40 s–50 s
6			50 s–70 s
7			70 s–80 s
8	Reintubated		60 s–80 s
9	Extubated, reintubated	Start, 6 pm	70 s–80 s
10			30 s–60 s
11	ECG: sinus bradycardia		40 s–50 s
12	ECG: bigeminy		30 s–50 s
13	Rhythm strip: bigeminy, PVCs	Last dose, 6 am	30 s–60 s
14	Tracheostomy		60 s–80 s
15			60 s–80 s
16			70 s–80 s
17			70 s-80 s
28	Discharged home with tracheostomy collar		80 s

**Table 2:** Naranjo Adverse Drug Reaction Probability Scale.

Question	Yes	No	Do not know	Score
Are there previous conclusive reports on this reaction?	+1	0	0	+1
Did the adverse event appear after the suspected drug was administered?	+2	−1	0	+2
Did the adverse reaction improve when the drug was discontinued or a specific antagonist was administered?	+1	0	0	+1
Did the adverse event reappear when the drug was re-administered?	+2	−1	0	+2
Are there alternative causes (other than the drug) that could on their own have caused the reaction?	−1	+2	0	+2
Did the reaction appear when a placebo was given?	−1	+1	0	0
Was the drug detected in the blood (or other fluids) in concentrations known to be toxic?	+1	0	0	0
Was the reaction more severe when the dose was increased or less severe when the dose was decreased?	+1	0	0	0
Did the patient have a similar reaction to the same or similar drugs in any previous exposure?	+1	0	0	0
Was the adverse event confirmed by any objective evidence?	+1	0	0	+1
			Total	+9
