# Supplementary material for: Bradycardia Associated with Steroid Use for Laryngeal Edema in an Adult: A Case Report and Literature Review
Source: Case Rep Cardiol. 2016 Nov 24;2016:9785467. doi: 10.1155/2016/9785467 (PMC5143689; doi:10.1155/2016/9785467)

S[redacted], G[redacted]   ID:[redacted]   01-SEP-2015 10:40:44   VA Baltimore

Male   Caucasian
68in   345lb
Room:
Loc:2

Vent. rate	48	BPM
PR interval	184	ms
QRS duration	110	ms
QT/QTc	480/428	ms
P-R-T axes	27 36	51

Marked sinus bradycardia
Abnormal ECG
When compared with ECG of 28-AUG-2015 22:01, (unconfirmed)
No significant change was found

Technician:
Test ind:

Referred by: Confirmed By: MICHAEL MILLER

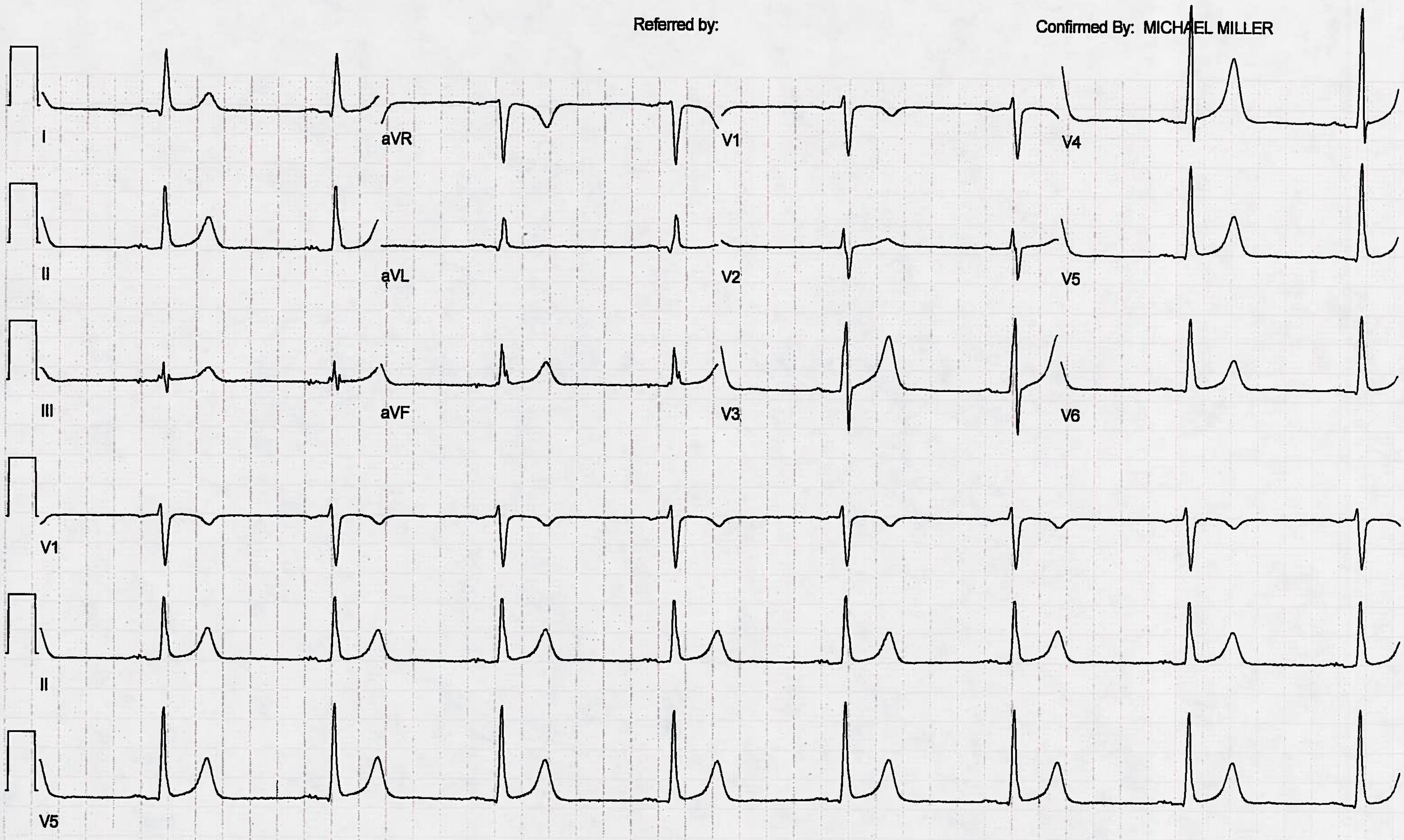

S[redacted]P, G[redacted] ID:[redacted] 01-SEP-2015 23:36:40 VA Baltimore

Male	Caucasian	Vent. rate	55	BPM
68in	345lb	PR interval	160	ms
Room:		QRS duration	94	ms
Loc:2		QT/QTc	478/457	ms
		P-R-T axes	23 29	37

Sinus bradycardia with frequent Premature ventricular complexes in a pattern of bigeminy
Otherwise normal ECG
When compared with ECG of 01-SEP-2015 10:40, (unconfirmed)
Premature ventricular complexes are now Present

Technician: FRANCES TAYMES
Test ind:

Referred by: Confirmed By: MICHAEL MILLER

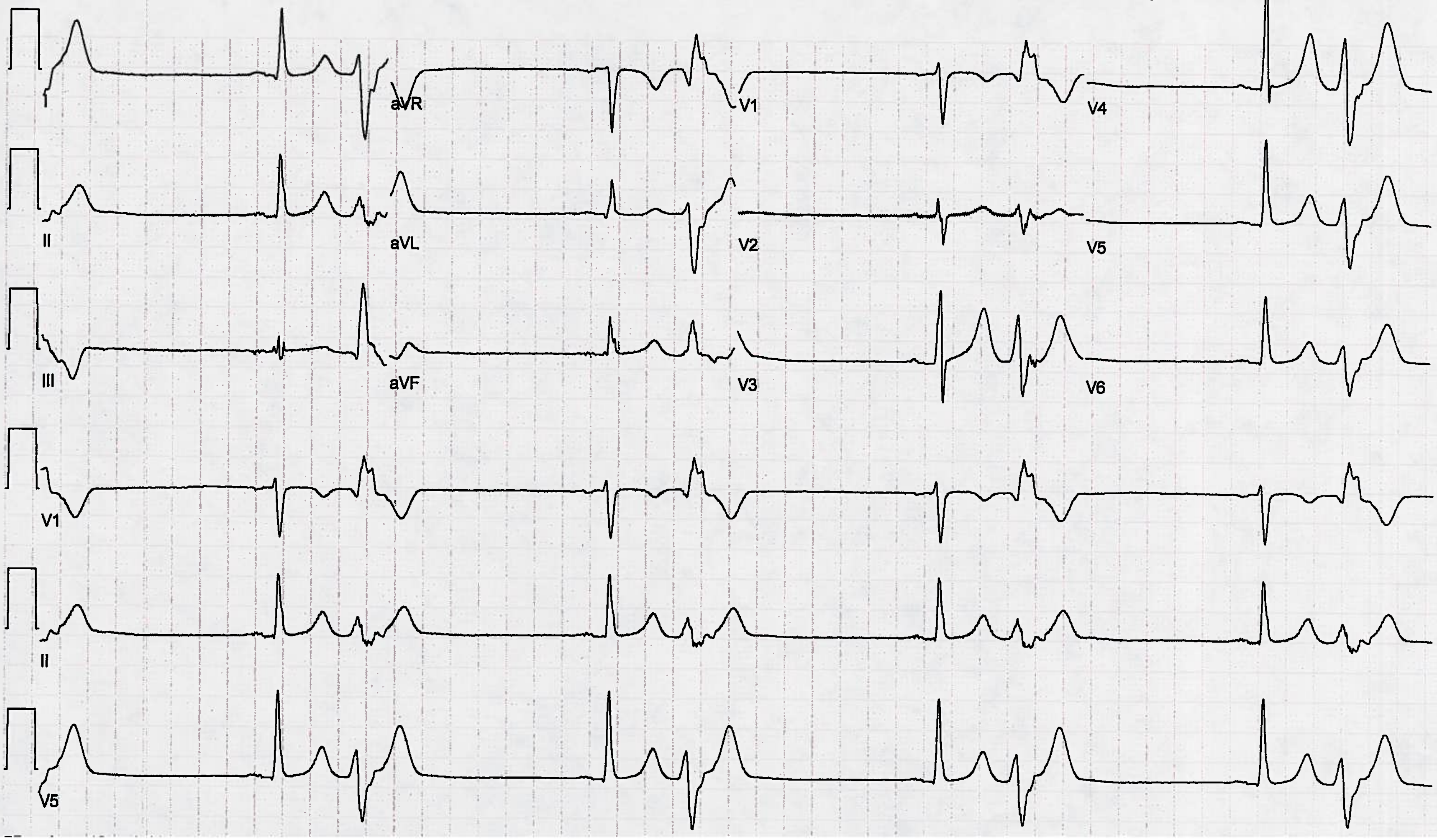

Supplement: Supplementary file 1 — Electrocardiograms obtained following steroid administration show the following:Sinus bradycardia; Sinus bradycardia with frequent premature ventricular contractions in a pattern of bigeminy. [file 9785467.f1.pdf]
